# Role of Teriparatide in Medication-Related Osteonecrosis of the Jaws (MRONJ)

**DOI:** 10.3390/dj4040041

**Published:** 2016-11-09

**Authors:** Yong-Dae Kwon, Deog-Yoon Kim

**Affiliations:** 1 Center for Refractory Jawbone Diseases, Department of Oral & Maxillofacial Surgery, Kyung Hee University Dental Hospital, Seoul 02447, Korea; 2 Department of Nuclear Medicine, Kyung Hee University Hospital, Seoul 02447, Korea; deogyoon@empal.com

**Keywords:** MRONJ, bisphosphonate, ONJ, teriparatide, parathyroid hormone

## Abstract

While the optimal treatment concept of medication-related osteonecrosis of the jaws (MRONJ) is still in debate, several adjunct therapies have been introduced. Among these adjunctive measures, recombinant human parathyroid hormone (rhPTH, teriparatide) seems to be the most promising treatment modality. Several studies have presented the beneficial effect of short-term teriparatide; they have shown an improved level of bone markers and radiographic evidence of bone healing. Although clinical validation by a controlled trial with prospective design would be essential, short-term teripratide therapy would be a good treatment option for MRONJ patients with impaired bone remodeling.

## 1. Introduction

Bone tissue undergoes an incessant formation and resorption process which is called remodeling. During the postmenopause period, bone loss and structural weakening can be deepened. To prevent excessive bone loss, antiresorptive therapy can reduce the intensity of such remodeling. Bone mineral density (BMD) is increased because resorption cavities present at the start of the treatment can be refilled with fewer new concomitant resorption cavities.

Since the first report of medication-related osteonecrosis of the jaws (MRONJ), dental surgeries in osteoporotic patients have been a key issue.

Teriparatide is a human recombinant peptide consisting of 1-34 amino acids of human parathyroid hormone which has 84 amino acids. The first 34 amino acids of human recombinant parathyroid hormone (rhPTH) have been approved for severe osteoporosis treatment. Among many osteoporosis regimens, the 1-34 rhPTH is believed to be the only anabolic agent. Parathyroid hormone is like a double-edged sword. It has both an anabolic and a catabolic effect on the bone, and the timing in which the exposure to this hormone happens is the critical determinant in the mode of action. Persistent increased PTH will deplete bone mass but intermittent exposure to PTH will activate more osteolastic activity than osteoclastic activity. The net effect of stimulating new bone formation may explain the increase of the bone mass. In fact, the molecular mechanism of the anabolic effect of PTH is unclear [1].

The treatment modalities of MRONJ have evolved and adjunctive therapies have been introduced. Among the adjunct therapies, such as hyperbaric oxygen, teriparatide is thought to be the most investigated modality since it was first reported [2]. Recently, there was a basic study showing potential therapeutic effect of geranylgeraniol [3]. Use of rhPTH(1-34) can enhance bone regeneration potential in elderly patients and we were able to see improved bone markers in our MRONJ patients. Because the use of rhPTH(1-34) can improve the whole body bone architecture, there is a concern regarding the risk of osteoporotic fracture that might result from a drug holiday.

In this literature review, current knowledge of the use of teriparatide for MRONJ treatment will be reviewed. The literature search was carried out by using Pubmed and the keywords were teriparatide, bispohosphonate osteonecrosis and parathyroid hormone. Only clinical reports were included (Table 1).

## 2. Adverse Action of Bisphosphonates to the Jaws

Since the first report of bisphosphonate-related osteonecrosis of the jaws, osteonecrosis of the jaws is a rare complication and can be a debilitating chronic disease, which may lead to poor oral intake. Although the possible pathogenesis of the ONJ has been extensively investigated, the relationship between the ONJ and bisphosphonate has not been fully proven. The estimates of ONJ prevalence at this moment have ranged from 0.001% to 0.01% among oral bisphosphonate-treated populations, largely from data from Australia and Germany [4]. From the PROBE study in 2010, they estimated a frequency of ONJ of 28 per 100,000 person-years of treatment [5].

It is quite well known that bisphosphonate suppresses bone remodeling and this may impair the repair damage from dental surgery involving the alveolar bone. In addition, bisphosphonate may affect angiogenesis and may produce soft tissue toxicity [6,7]. Compromised function of the neutrophil might also be a potential reason for ONJs [8] and bisphosphonate might impair neutrophil chemotaxis, possibly contributing to the development of MRONJ lesions [9].

## 3. Result and Discussion

### 3.1. Teriparatide and Its Anabolic Effects

The intermittent infusion of low-dose PTH results in anabolic effects on the bone [10]. These anabolic actions of this hormone involve direct effects on osteoblast lineage and indirect effects through the regulation of some selected growth factors, which are skeletally related (e.g., IGF-I) and growth factor antagonists such as sclerostin [10].

Antiresportive therapy using bisphosphonate has been most popular among many treatment regimens. Basically, antiresorptive therapy maintains bone mass by preventing the drainage of bone minerals. Teriparatide is approved in the United States for the treatment of osteoporosis in postmenopausal women and in men who are at high risk of bone fracture. Unlike the antiresportive agents, teriparatide is the only anabolic agent approved by the FDA for osteoporosis treatment and its bone formation ability has been clinically validated via RCTs [11,12]. The outcomes of teriparatide and alendronate have been contrasted in an RCT and those two distinct options increased BMD by opposite mechanisms [12].

Unlike antiresorptive regimens, teriparatide increases bone formation earlier and to a greater degree than resorption, leading to a positive bone balance, which means an increase in bone mineral density (BMD), an improvement in the microarchitecture and strength, and a decrease in fracture incidence [13,14]. Teriparatide therapy increases the rate of bone formation by the first month of the treatment and this can be explained by an ‘anabolic window’ [15]. According to the recent RCT which is called DATA (Denosumab and Teriparatide Administration) extension study, the anabolic-catabolic gap seemed to be narrower but the duration seemed to be longer [16]. 

### 3.2. Possible Utilities of Teriparatide in Oral and Maxillofacial Region

Basically, teriparatide has been approved for the treatment of post-menopausal osteoporosis with high risk of fractures, idiopathic or hypogonadal osteoporosis in males with high risk of fractures and steroid-induced osteoporosis. Benefits of PTH on vertebral and appendicular bone density have been well-investigated; however, craniofacial skeletons’ response to PTH similarly is still not clear and should be investigated more. Since the developmental origins of craniofacial bone formation are different when compared with those of the other skeletal sites, the mechanisms of PTH action may vary based on location.

As for dental implications, teriparatide administration seemed to be beneficial for treating chronic periodontitis, showing the repair potential for localized bone defects in the jaws [17]. In this trial, application of teriparatide combined with periodontal surgery, compared to the periodontal surgery only, showed the improved clinical and radiographic outcomes. Given that linear bone gain after ordinary periodontal surgery without regenerative measure should be minimal, improved regeneration of periodontal bone with teriparatide injections may be encouraging in terms of its possible utility in periodontal treatment [17].

For dental implant and ridge augmentation procedures, the effect of teriparatide can also be beneficial. Although implants can be successfully integrated in most people, certain groups of patients may present higher failure rates due to their medical conditions. An animal study has shown a positive role of PTH for improving impaired wound healing capacity. Delayed wound healing was induced by daily injection of a steroid, and 1-mm defects were made in the ulnae of rabbits [18]. Teriparatide in conjunction with bone grafts could enhance alveolar bone formation in several animal studies [19,20].

### 3.3. Background of Using Teriparatide for MRONJ Patients

MRONJ which is a rare complication of long-term antiresorptive therapy has been a major concern in dentistry. The treatment course has often been frustrating and the outcome has also been often suboptimal. Regarding the treatment modalities of MRONJ, conservative treatment such as oral antibacterial rinse and antibiotics has been prevailing over time, but surgical treatment has simply been regarded as successful with gaining more experience in this particular disease [21,22]. 

The anabolic characteristic of teriparatide has prompted us to use this peptide for ONJ patients. Recently, many clinical studies have been published with favorable results in treating MRONJ [2,23,24,25,26,27,28,29,30]. Basically, teriparatide can enhance bone formation before the stimulation of bone resorption process and using teriparatide may promote bone repair in and around the defect of the lesions. In fact, MRONJ is a complication of long-term anti-resorptive therapy blocking the action of osteoclasts but the target of the teripatatide therapy is to activate osteoblstic action. According to one of our studies, serum osteocalcin study of the patients presented significantly depressed values [31]. Although bisphosphonates do not directly affect the bone formation mechanism, impaired bone healing can be expected in the elderly population and this condition can be stratified by medical co-morbidities such as diabetes and rheumatism. The suppression of osteoclasts by bisphosphonates not only disturbs bone resorption process but may also exert negative effects on bone formation because the interaction between the two cellular populations is disrupted. Bone formation capacity in MRONJ patients can be an important parameter of the treatment outcome. Lee et al. [32] investigated possible clinical parameters that may affect the treatment outcome of MRONJ and higher level of bone specific alkaline phosphatase at baseline showed a better prognosis. Bone formation markers respond immediately to intermittent administration of teriparatide in osteoporotic females. Considering such research outcomes, recovering the osteoclastic activity cannot solely ensure proper healing for this special category of patients.

### 3.4. Teriparatide as a Treatment Modality of MRONJ

Looking at the current therapy of MRONJ, the treatment modalities have been nothing but traditional so far. Among several treatment options, surgery is the most reliable one, especially for MRONJ patients whose indication of bisphosphonate is osteoporosis [21]. The most popular treatment modality comprises: a drug holiday, oral rinse, antibiotics and surgery. Given that the surgical method is one of the most efficient treatment options, surely combined with conservative measures, promotion of the surgical wound might be beneficial to proper healing. In the context of the promotion of surgical wound healing, the application of teriparatide would be reasonable.

Because of the anabolic action of teriparatidfe, it has been anecdotally suggested as a beneficial treatment option in situations that need enhancement of bone remodeling. Teriparatide has clinically demonstrated greater regaining of alveolar bone defects and accelerated osseous wound healing in the oral cavity of chronic periodontitis [17]. From teriparatide therapy, improvement of suppressed bone markers was presented in some of the literatures [24,25,30] and this finding provided potential roles to facilitate bone healing. The treatment duration of teriparatide may vary depending on the decision of prescribing physicians. In fact, there is no generally accepted treatment course. In one study, 1–3 months of teriparatide therapy was provided along with the surgical debridement [25]. In another study, 6 months of teripratide therapy was carried out for the MRONJ patients who were unresponsive to conventional treatments [24]. Longer administration of teriparatide (about 6 months) would be beneficial to the healing of the lesions but financial burden to the patients might be an obstacle in a real clinical situation. Along with surgery, short-term teriparatide therapy might be reasonable in terms of the initial healing promotion of the lesions after the surgical debridement. However, in a letter, a non-responsive case to teriparatide was presented and anti-rheumatic drugs were attributed to one possible reason because of their effect on healing impairment [26].

It is quite well-known that bisphodphonates have an anti-angiogenic action [33,34,35] and this can be considered as one of the etiologic factors of MRONJ. According to a recent research, teriparatide was assumed to enhance angiogenesis, implying that teriparatide therapy may be also beneficial for the healing via increased angiogenesis activity [36]. However, in some animal studies, experimentally induced MRONJ-like lesions did not show the angiogenesis defects [37,38]. Recombinant human monoclonal antibody like bevacizumab shows antitumor effects via binding to vascular endothelial growth factor, thus inhibiting angiogenesis, and it was recently reported to be possibly associated with MRONJ cases [39]; however, teriparatide therapy for those patients is inappropriate.

Vitamin D is essential to bone metabolism and this is frequently prescribed to osteoporotic patients with oral calcium preparations. In the one of the studies about the application of teriparatide to MRONJ patients, the researchers found that the serum vitamin D level at the baseline was thought to be an influencing factor. In their study, better clinical outcome was noted in a group showing a higher level of serum vitamin D [24] and this may imply that lower level of vitamin D can deter bone healing. Actually, an optimal level of vitamin D is considered important for mineralization [40]. Supplemental vitamin D with calcium may be considered for the patients presenting a markedly low level of serum vitamin D. Many studies suggest that bisphosphonates, which potentially disturb bone resorption process, may substantially affect the initial response to teriparatide [41]. Therefore, some clinicians claimed that a certain period of washout phase may be necessary before teriparatide administration [42]. Meanwhile, teriparatide and vitamin D can reverse the antiresorptive effect of bisphosphonates [43].

A drug holiday is believed to be one of the core components of MRONJ treatment, despite complications like osteoporotic fractures in the vertebrae, femur and pelvis. There have been some reports showing that a drug holiday can be acceptable without a significant rise of the complication rate [44,45]. However, the increased risk of osteoporotic fractures which may be fatal should not be ignored, because the safety issue of the drug holiday should be further investigated. Moreover, some clinicians claimed drug holiday significantly increased the risk of fractures [46]. Considering the possible risk of osteoporotic fractures during drug holiday, teriparatide therapy may have dual benefits for MRONJ patients, promoting bone healing of the surgical wound of MRONJ patients and increasing bone density. Therefore, concerns regarding a drug holiday can be minimized by teriparatide which is also a therapeutic agent for osteoporosis.

Currently, most of the literature about teriparatide therapy consists of case studies or retrospective studies. In order to validate the possible use of teriparatide, a controlled trial with prospective design should be conducted. Many MRONJ patients may have complex medical co-morbidities, so dentists or surgeons should communicate with their prescribing physicians when a treatment plan for MRONJ is set up.

## 4. Conclusions 

While treatment modalities for MRONJ have been evolved and surgical treatment combined with conservative measures has been quite successful, an anabolic approach using teriparatide may be beneficial to these patients with MRONJ who are often unresponsive to conventional treatment. Teriparatide can facilitate bone wound healing.

## Figures and Tables

**Table 1:** Human studies on teriparatide application for medication-related osteonecrosis of the jaws (MRONJ) treatment.

Reference	Patient No.	BP Indication	BP Route	Duration (Years)	Trigger Event	Staging	Duration of TPTD	Outcome
Harper and Fung (2007) [2]	1	OP	Oral	1	ext	2	3 months	Favored
Cheung and Seeman (2010) [23]	1	OP	Oral	10	ext	2	8 weeks	Favored
Lau and Adachi (2009) [27]	1	OP	Oral/IV	10	ext	2	2 months	Favored
Tsai et al. (2010) [47]	1	NA	Oral	4	imp	3	5 months	Favored
Narongroeknawin et al. (2010) [29]	1	OP	Oral	5	imp	1	4 months	Favored
Lee et al. (2011) [28]	1	OP	Oral	5	ext	2	6 months	Favored
Kwon et al. (2012) [25]	6	OP	Oral	3 to 8	NA	2 or 3	1 to 3 months	Favored
Narvarez et al. (2012) [26]	1	OP	NA	2.7	spon	NS	8 months	Unfavored
Kim et al. (2014) [24]	15	OP	Oral/IV	3	ext/imp/spon	2 or 3	6 months	Favored
Kakehashi et al. (2015) [30]	10	NA	Oral	4.3	ext/perio	2 or 3	4 to 24 months	Favored

OP = osteoporosis; NA = not available; BP = bisphoshponate; TPTD = teriparatide; ext = extraction; imp = implant; spon = spontaneous; perio = periodontitis.

## Abbreviations

The following abbreviations are used in this manuscript:

MRONJ	Medication related osteonecrosis of the jaw
rhPTH	Recombinant human parathyroid hormone
